# GADD34-mediated dephosphorylation of eIF2α facilitates pseudorabies virus replication by maintaining de novo protein synthesis

**DOI:** 10.1186/s13567-021-01018-5

**Published:** 2021-12-20

**Authors:** Ting Zhu, Xueli Jiang, Hangkuo Xin, Xiaohui Zheng, Xiaonuan Xue, Ji-Long Chen, Baomin Qi

**Affiliations:** Key Laboratory of Fujian-Taiwan Animal Pathogen Biology, College of Animal Science, Fujian Agriculture and Forestry University, Fuzhou, China

**Keywords:** host translation, eIF2α, phosphorylation, PRV, GADD34

## Abstract

Viruses have evolved multiple strategies to manipulate their host’s translational machinery for the synthesis of viral proteins. A common viral target is the alpha subunit of eukaryotic initiation factor 2 (eIF2α). In this study, we show that global protein synthesis was increased but the eIF2α phosphorylation level was markedly decreased in porcine kidney 15 (PK15) cells infected with pseudorabies virus (PRV), a swine herpesvirus. An increase in the eIF2α phosphorylation level by salubrinal treatment or transfection of constructs expressing wild-type eIF2α or an eIF2α phosphomimetic [eIF2α(S51D)] attenuated global protein synthesis and suppressed PRV replication. To explore the mechanism involved in the inhibition of eIF2α phosphorylation during PRV infection, we examined the phosphorylation status of protein kinase R-like endoplasmic reticulum kinase (PERK) and double-stranded RNA-dependent protein kinase R (PKR), two kinases that regulate eIF2α phosphorylation during infection with numerous viruses. We found that the level of neither phosphorylated (p)-PERK nor p-PKR was altered in PRV-infected cells or the lungs of infected mice. However, the expression of growth arrest and DNA damage-inducible protein 34 (GADD34), which promotes eIF2α dephosphorylation by recruiting protein phosphatase 1 (PP1), was significantly induced both in vivo and in vitro. Knockdown of GADD34 and inhibition of PP1 activity by okadaic acid treatment led to increased eIF2α phosphorylation but significantly suppressed global protein synthesis and inhibited PRV replication. Collectively, these results demonstrated that PRV induces GADD34 expression to promote eIF2α dephosphorylation, thereby maintaining de novo protein synthesis and facilitating viral replication.

## Introduction

Viruses lack biosynthetic capabilities and depend on the host machinery to synthesize viral proteins. Consequently, viruses must effectively seize control of host translation factors and associated regulatory networks [1]. In the host, translation is primarily regulated in the initiation phase by a process that involves ribosome recruitment and the production of mRNA [2]. Eukaryotic initiation factor 2 (eIF2), which is required for translation initiation in most eukaryotes, is a trimeric complex composed of α, β, and γ subunits. When bound to GTP, the eIF2 complex recruits Met-tRNAi and the 40S ribosomal subunit to initiate mRNA translation [3, 4]. When the initiation phase is completed, GTP is hydrolysed to GDP, leading to the release of inactive eIF2-GDP from the ribosome. eIF2-GDP can be recycled into its active form (eIF2-GTP) through the activity of the guanine exchange factor eIF2B, leading to another round of initiation. However, in response to different types of stress, the α subunit of eIF2 (eIF2α) can be phosphorylated on serine 51, which inhibits the dissociation of eIF2B from eIF2, thereby preventing the recycling of GTP on the eIF2 complex and resulting in global inhibition of protein synthesis [5, 6]. As a countermeasure, viruses either inhibit the activation of eIF2α kinase or increase the rate of eIF2α dephosphorylation to overcome the inhibitory effects of eIF2α phosphorylation, ultimately maintaining viral protein synthesis [7].

eIF2α is known to be phosphorylated by at least four different kinases, namely, general control nonderepressible-2 (GCN-2), protein kinase R-like endoplasmic reticulum kinase (PERK), double-stranded RNA (dsRNA)-dependent protein kinase R (PKR), and haem-regulated inhibitor (HRI), in response to a variety of stress conditions [6, 7]. Among these kinases, PERK and PKR play critical roles in viral infection via phosphorylation of eIF2α. PKR is normally present in cells in an inactive state. Following activation by dsRNA, it undergoes autophosphorylation, which activates its eIF2α kinase activity [8, 9]. PKR activation is an antiviral response that reduces the translation of viral proteins in infected cells [10]. For instance, Newcastle disease virus (NDV) infection activates PKR, which inhibits NDV replication via eIF2α phosphorylation during late infection stages [11], while during herpes simplex virus type 1 (HSV-1) infection, activated PKR phosphorylates eIF2α, thereby inhibiting protein synthesis and, consequently, viral replication [12, 13]. However, viruses have evolved many countermeasures to inhibit the activation of PKR or the phosphorylation of eIF2α [14]. For example, porcine reproductive and respiratory syndrome virus (PRRSV) inhibits PKR activation and eIF2α phosphorylation during the early stages of infection, and this inhibition is thought to be essential for the initiation of viral replication [15]. PERK is an endoplasmic reticulum (ER) transmembrane protein that specifically phosphorylates eIF2α when activated through the unfolded protein response (UPR), thereby suppressing protein synthesis [16, 17]. Some viruses can selectively activate or repress the UPR to facilitate their own replication via the PERK pathway, as seen with HSV-1 infection [13]. It is also known that the E2 protein of hepatitis C virus binds to PERK and inhibits its activation, thereby reversing PERK-mediated translational repression and promoting persistent viral infection [18].

Both protein phosphatase 1 (PP1) and protein phosphatase 2 (PP2A) have been reported to dephosphorylate eIF2α [19]. PP1 regulates a number of cellular functions through the interaction of its catalytic subunit (PP1c) with many regulatory partners [20]. One well-established example is growth arrest and DNA damage-inducible protein 34 (GADD34), which physically interacts with PP1c, leading to enhanced eIF2α dephosphorylation both in vitro and in vivo [21]. The role of GADD34 in host–virus interactions has been widely documented. For instance, in mouse embryonic fibroblasts, vesicular stomatitis virus (VSV) infection can induce GADD34 expression, which, in turn, suppresses viral replication by dephosphorylating TSC2 in the mTOR pathway [22]. In another example, the E6 oncoprotein of human papillomavirus type 18 reportedly associates with the GADD34/PP1 holophosphatase complex, which mediates translational recovery and facilitates eIF2α dephosphorylation [23]. Finally, infectious bronchitis virus (IBV), a chicken coronavirus, induces GADD34 expression, thereby promoting eIF2α dephosphorylation and maintaining de novo protein synthesis in infected cells [1].

In this study, we report that pseudorabies virus (PRV), a member of the Alphaherpesvirinae subfamily within the family Herpesviridae [24], increased global translation and GADD34 expression and suppressed eIF2α phosphorylation. An increase in the eIF2α phosphorylation level by salubrinal treatment or transfection of constructs expressing wild-type eIF2α or an eIF2α phosphomimetic [eIF2α(S51D)] attenuated global protein synthesis and suppressed PRV replication. In contrast, inhibition of PP1 activity by okadaic acid (OA) treatment or knockdown of GADD34 by small interfering RNA regulated eIF2α phosphorylation as well as PRV replication.

## Materials and methods

### Cell culture and viral infection

Porcine kidney 15 (PK15) cells (ATCC CCL-33) and Madin-Darby canine kidney (MDCK) cells (ATCC CRL-2935) were maintained in Dulbecco’s modified Eagle’s medium (DMEM) (Gibco-BRL, Gaithersburg, MD, USA) supplemented with 10% foetal bovine serum (FBS) (Gibco-BRL), 100 μg/mL streptomycin, and 100 IU/mL penicillin. The PRV strain Min-A was propagated in MDCK cells, and the viral load was titrated in PK15 cells using a TCID_50_ assay, as previously described [25].

PK15 cells were infected with PRV Min-A at a multiplicity of infection (MOI) of 0.01. After absorption for 1 h at 37 °C, the cells were washed three times with PBS to remove unbound virus and were then incubated with fresh medium (supplemented with 2% FBS) at 37 °C for the indicated times.

### Mouse challenge experiment

Specific pathogen-free BALB/c mice were obtained from Wushi Animal Center (Shanghai, China). Female mice (5 to 6 weeks old) were intraperitoneally inoculated with 1 × 10^6^ plaque-forming units (PFUs) of PRV or 100 µL of sterile PBS. At the indicated times post-infection (pi), mice were euthanized, and their lungs were harvested for further analysis by Western blot and RT–qPCR.

### Antibodies and reagents

Antibodies against phosphorylated (p)-eIF2α (Ser51) (ab32157), p-PKR (Thr451) (ab81303), and total PKR (ab32052) were purchased from Abcam (Cambridge, MA, USA). The antibody against total eIF2α (sc-11386) was purchased from Santa Cruz Biotechnology (Dallas, TX, USA). The monoclonal antibody clone 16F8 against p-PERK (Thr980) (#3179) was purchased from Cell Signaling Technology (Danvers, MA, USA). Antibodies against PERK (20582-1-AP), GADD34 (10449-1-AP), and β-actin (20536-1-AP) were obtained from Proteintech Group (Chicago, IL, USA). The antibody against PRV (PA1-081) was obtained from Invitrogen (Carlsbad, CA, USA), the monoclonal antibody clone 12D10 against puromycin (MABE343) was obtained from Sigma–Aldrich (St. Louis, MO, USA), and the HRP-conjugated IgG secondary antibody was purchased from Jackson ImmunoResearch Laboratories (West Grove, PA, USA). Thapsigargin (Tg) (67526-95-8), salubrinal (324895), and puromycin (540222) were purchased from Sigma–Aldrich. The PP1/PP2A inhibitor okadaic acid (OA) (GC16958) was purchased from GlpBio (Montclair, CA, USA). Lipofectamine 2000 was purchased from Invitrogen.

### Western blot analysis

Cell lysates were prepared, and Western blotting was performed as previously described [26]. Briefly, equal amounts of protein were separated by SDS–PAGE, transferred onto nitrocellulose membranes (Millipore, Billerica, MA, USA), blocked with 5% nonfat dry milk in TBS (20 mM Tris–HCl, pH 7.4; 150 mM NaCl) at 37 °C for 1 h, and incubated at 4 °C overnight with different primary antibodies. After washing three times with TBS, the membranes were incubated with the HRP-conjugated secondary antibody for 1 h and were then washed three times with TBS. The protein bands were visualized with a FluorChem M Imaging System (ProteinSimple, CA, USA). Membranes were stripped with stripping buffer (10 mM β-mercaptoethanol; 2% SDS; 62.5 mM Tris, pH 6.8) at 55 °C for 30 min before being reprobed with another antibody.

### Puromycin labelling and chemical treatment

PK15 cells were infected with PRV or mock infected and were then labelled with 10 μg of puromycin for 1 h at different times pi. After puromycin labelling, all cells were washed three times with precooled PBS and lysed in lysis buffer. Equivalent amounts of protein extracts were subjected to Western blot analysis using the anti-puromycin antibody clone 12D10.

Tg, salubrinal, and OA were dissolved in DMSO. PK15 cells were incubated with PRV in cell culture medium containing 1 µM Tg and harvested after 24 h. To inhibit eIF2α dephosphorylation, 100 µM salubrinal was added to cells immediately after PRV infection. After 23 h of incubation, the cells were labelled with puromycin.

To inhibit PP1/PP2A activity, PK15 cells were infected with PRV and incubated at 37 °C for 8 h before the addition of OA at the indicated concentrations. The cells were then incubated for another 15 h prior to puromycin labelling.

### Recombinant plasmid construction and plasmid transfection

The eIF2α sequence was generated by PCR amplification of the corresponding cDNA obtained from PK15 cells. Amplified fragments were purified and were then inserted into the pCMV-HA vector to construct the eIF2α (wild-type) plasmid using a pEASY-Basic Seamless Cloning and Assembly Kit (TransGen Biotech, Beijing, China). The eIF2α(S51D) and eIF2α(S51A) mutant sequences were generated by site-directed mutagenesis. The recombinant plasmids were confirmed by sequencing.

The GADD34 short hairpin RNA (shRNA) expression plasmid pGPH1/GFP/Neo-shRNA-GADD34 (shGADD34) and the negative control plasmid pGPH1/GFP/Neo-shRNA-NC (shNC) containing a scrambled shRNA sequence were obtained from GenePharma (Shanghai, China). The shRNA sequences are listed in Table 1.

**Table 1:** **List of primers used in the study**

Primer name	Primer sequence (5’-3’)
PRV-*gE* F	CCACTCGCAGCTCTTCTCG
PRV-*gE* R	CAGTCCAGCGTGGCAGTAAA
*β-actin* F	CGGCATCCACGAAACTACCT
*β-actin* R	GCCGTGATCTCCTTCTGCAT
eIF2α F	GTCGACCGAGATCTCTCGAG ATGCCGGGTCTGAGTTGTAGAT
eIF2α R	CGCGGCCGCGGTACCTCGAGTT AATCTTCAGCTTTGGCTTCC
eIF2α(S51A) F	CTTCTTAGTGAGCTCGCCAG AAGGCGTATCC
eIF2α(S51A) R	GGATACGCCTTCTGGCGAG CTCACTAAGAAG
eIF2α(S51D) F	CTTCTTAGTGAGCTCGACAG AAGGCGTATCCG
eIF2α(S51D) R	CGGATACGCCTTCTGTCGAG CTCACTAAGAAG
shNC	TTCTCCGAACGTGTCACGT
shGADD34-1#	GGCTGGAGAAGCTGTAAATAA
shGADD34-2#	GAGCCCGGAAGTGAATTTATG
shGADD34-3#	GTGGCTGAGTTGAAGTAGTTT
shGADD34-4#	GGCCATCTATTTACCTGGAGA

PK15 cells were seeded into 12-well plates and grown to 70–80% confluence. The pCMV-HA, eIF2αwt, eIF2α(S51D), and eIF2α(S51A) vectors, as well as shGADD34 and shNC, were transfected into cells using Lipofectamine 2000 according to the manufacturer’s instructions. Twenty-four hours post-transfection, cells were infected with PRV. The cells and supernatants were then harvested at the indicated times for further analysis.

### Plaque formation assay

PK15 cells were seeded into 6-well plates and were then infected with the supernatants of virus-infected cells for 1 h. After washing with PBS, DMEM containing 2% FBS and 1% methylcellulose (Sigma–Aldrich) was added to the cells, which were then incubated at 37 °C for an additional 72 h. Visible plaques were counted to determine the viral titre.

### Reverse transcription-quantitative PCR (RT–qPCR)

Total RNA was extracted from cells or tissues using TRIzol reagent (Invitrogen) according to the manufacturer’s instructions. Isolated RNA (4 μg) was reverse transcribed into cDNA using M-MLV RT (Promega, Madison, WI, USA) according to the manufacturer’s instructions. qPCR was performed using 2 × TranStar Green qPCR SuperMix (TransGen Biotech). Beta-actin was used as the reference gene for internal normalization. To assess PRV replication, the gE gene of PRV was used as the standard for the PRV genome. Primers specific for PRV-gE, GADD34, and β-actin were designed using Primer Premier 5 (Premier Biosoft International, Palo Alto, CA, USA) (Table 1). The RT–qPCR data are shown as normalized expression ratios, which were automatically calculated by the LightCycler system (Roche, Basel, Switzerland) using the ΔΔCT method.

### Ethics statement

The animal protocol used in this study was approved by the Research Ethics Committee of the College of Animal Science, Fujian Agriculture and Forestry University, Fujian, China (permit no. PZCASFAFU2014002). The procedures were conducted in accordance with the approved guidelines.

### Densitometry

Band intensities were quantified using ImageJ software (U.S. National Institutes of Health, Bethesda, MD, USA).

### Statistical analyses

Data are presented as the mean ± standard deviation of three independent experiments. Parametric data were compared using Student’s *t* test or one-way analysis of variance followed by Tukey’s post-hoc test using SPSS software (SPSS Inc., Chicago, IL, USA) or GraphPad Prism 5 (GraphPad Software, La Jolla, CA, USA). *p* < 0.05 was considered to indicate a significant difference.

## Results

### PRV infection increased protein synthesis in PK-15 cells

**Figure 1:**
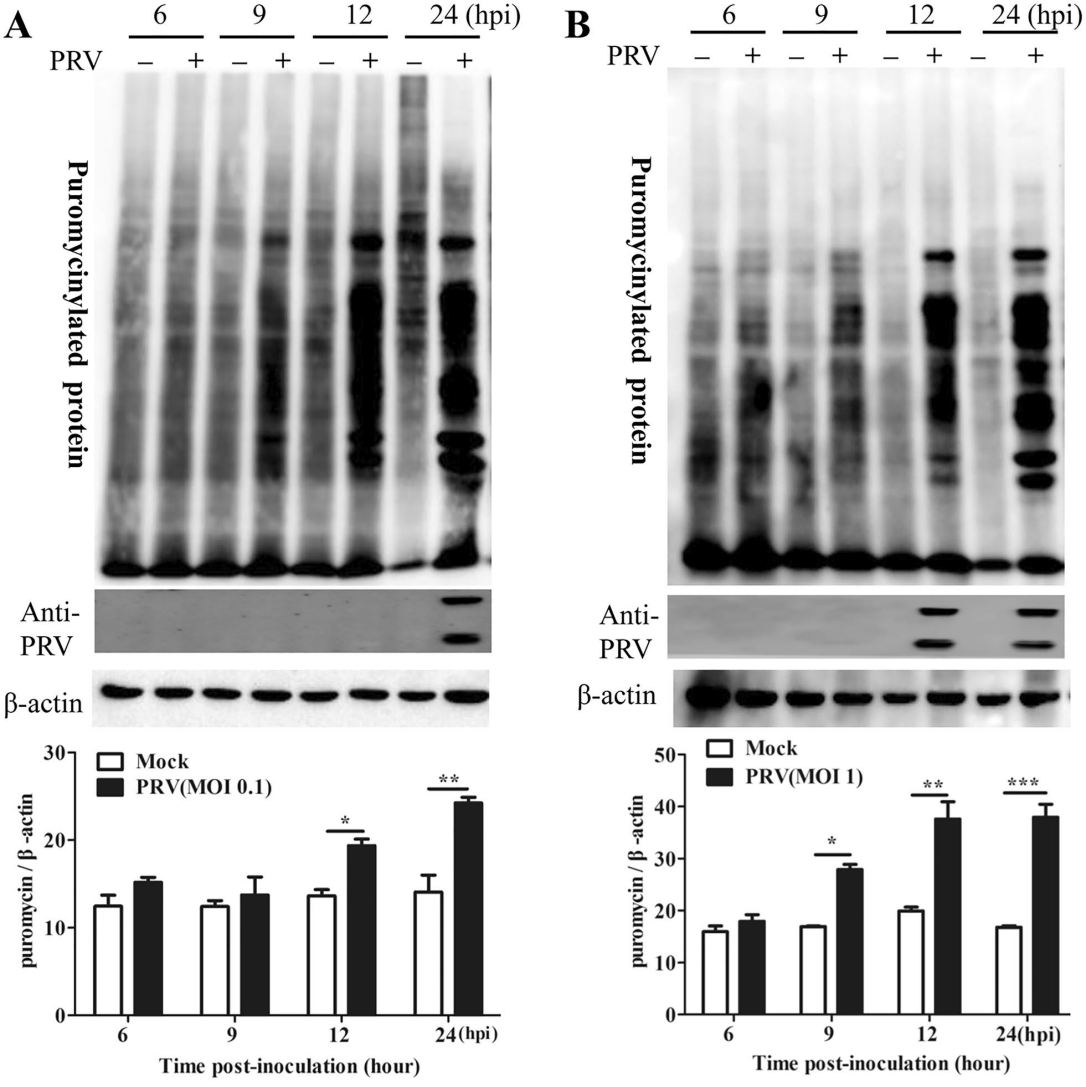
**Pseudorabies virus (PRV) infection increased global protein synthesis in PK15 cells.** Mock-infected PK15 cells and PK15 cells infected with PRV at an **A** MOI of 0.1 or **B** MOI of 1.0 were labelled with puromycin for 1 h at 5, 8, 11, and 23 hpi, and the cells were then harvested at 6, 9, 12, and 24 hpi. Cell lysates were subjected to Western blot analysis with the anti-puromycin mAb clone 12D10 to detect de novo protein synthesis. To monitor PRV replication, antibodies against PRV were used to detect PRV proteins. β-Actin was included in the Western blot analysis to document equivalent protein loading. The intensities of bands corresponding to puromycin-labelled proteins were determined by densitometry, and the protein synthesis rate is shown as the fold change after normalization to β-actin (bottom panels). The values are presented as the mean ± SD of triplicate experiments. * *p* < 0.05; ** *p* < 0.01; ***p < 0.001.

Viruses lack biosynthetic capabilities and depend on their host’s translational machinery to synthesize viral proteins [27]. To investigate host protein synthesis in response to PRV infection, PK15 cells were either mock infected or infected with PRV at different MOIs (0.1 or 1) and were then subjected to puromycin labelling for 1 h before being harvested at the indicated times. De novo protein synthesis was assessed by Western blot analysis using the anti-puromycin monoclonal antibody clone 12D10, which was used to detect newly synthesized puromycin-labelled proteins. The results showed that global protein synthesis remained unaffected up to 9 h post-infection (hpi); however, a significant increase in protein synthesis was observed from 12 to 24 hpi at an MOI of 0.1 (Figure 1A); densitometric analysis of the bands corresponding to puromycin-labelled proteins showed a 1.42–1.72-fold increase in the translation rate at 12–24 hpi in PRV-infected cells compared with mock-infected cells. In addition, new protein synthesis was significantly increased from 9 to 24 hpi in cells infected at an MOI of 1 (Figure 1B), with densitometric analysis of the bands corresponding to puromycin-labelled proteins showing a 1.65–2.26-fold increase in the translation rate at 9–24 hpi in PRV-infected cells compared with mock-infected cells. These findings indicated that PRV infection led to an increase in global translation.

### PRV infection suppressed eIF2α phosphorylation in vitro and in vivo

**Figure 2:**
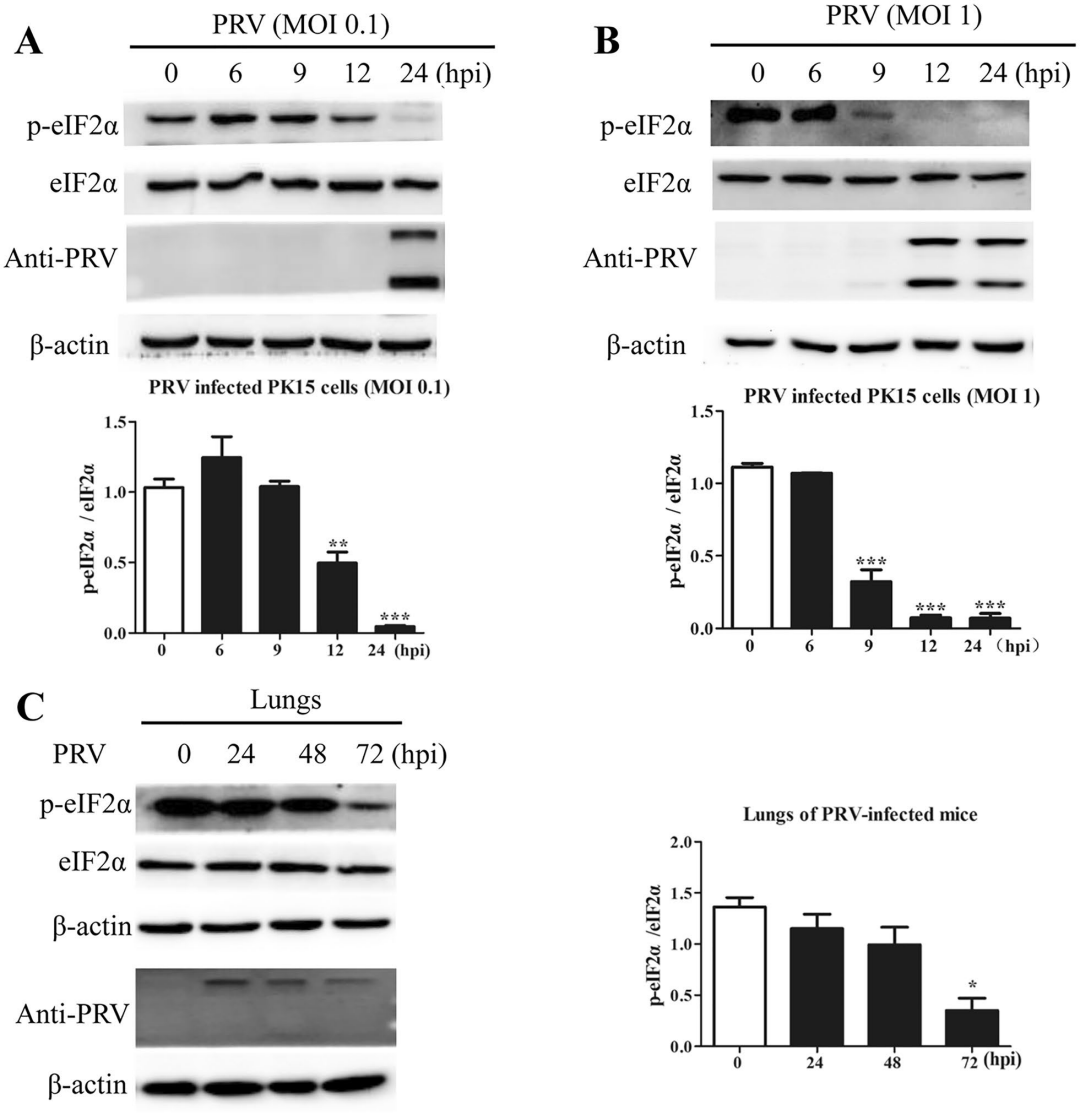
**PRV infection reduced the level of phosphorylated eIF2α in vitro and in vivo.** PK15 cells infected with PRV at an **A** MOI of 0.1 or **B** MOI of 1.0 were harvested and lysed at 0, 3, 6, 9, 12, and 24 hpi. The levels of p-eIF2α, eIF2α, and PRV proteins were determined by Western blot analysis. The intensities of the p-eIF2α bands were determined by densitometry, normalized to eIF2α, and shown as fold changes (bottom panel). The values are presented as the mean ± SD of triplicate experiments. ** *p* < 0.01; *** *p* < 0.001. **C** BALB/c mice were intraperitoneally inoculated with PRV (1 × 10^6^ PFU) and were then euthanized at 0, 24, 48, and 72 hpi. The levels of p-eIF2α, eIF2α, and PRV proteins in the lungs were determined by Western blot analysis. The intensities of the p-eIF2α bands were determined by densitometry, normalized to eIF2α, and shown as fold changes (right panel). The values are presented as the mean ± SD of triplicate experiments. * *p* < 0.05.

eIF2α is a key regulator of translation initiation, and its phosphorylation/dephosphorylation at Ser51 is a key determinant of the global translation rate [27]. Therefore, to determine the mechanisms that control de novo synthesis in PRV-infected cells, we used Western blot analysis to measure the relative protein levels of eIF2α and p-eIF2α in PK15 cells infected with PRV at different MOIs (0.1 or 1) and harvested at the indicated times. As shown in Figure 2A, the level of p-eIF2α declined at 12 hpi, and p-eIF2α was barely detectable at 24 hpi in cells infected at an MOI of 0.1, while in cells infected at an MOI of 1, the level of p-eIF2α was significantly reduced at 9 hpi, and p-eIF2α was barely detectable between 12 and 24 hpi (Figure 2B). The level of total eIF2α remained largely stable. We further found that the level of p-eIF2α decreased significantly at 72 hpi in the lungs of PRV-infected mice (Figure 2C). Taken together, these results indicated that PRV suppressed the phosphorylation of eIF2α both in vitro and in vivo. Owing to the loading of a large amount of each sample for protein analysis in this experiment and the prolonged exposure time, a relatively high level of p-eIF2α was detected in PK15 cells and lung tissue at 0 hpi. Indeed, a high basal level of p-eIF2 has also been reported in other studies [28–30].

### Increased phosphorylation of eIF2α inhibited PRV replication

**Figure 3:**
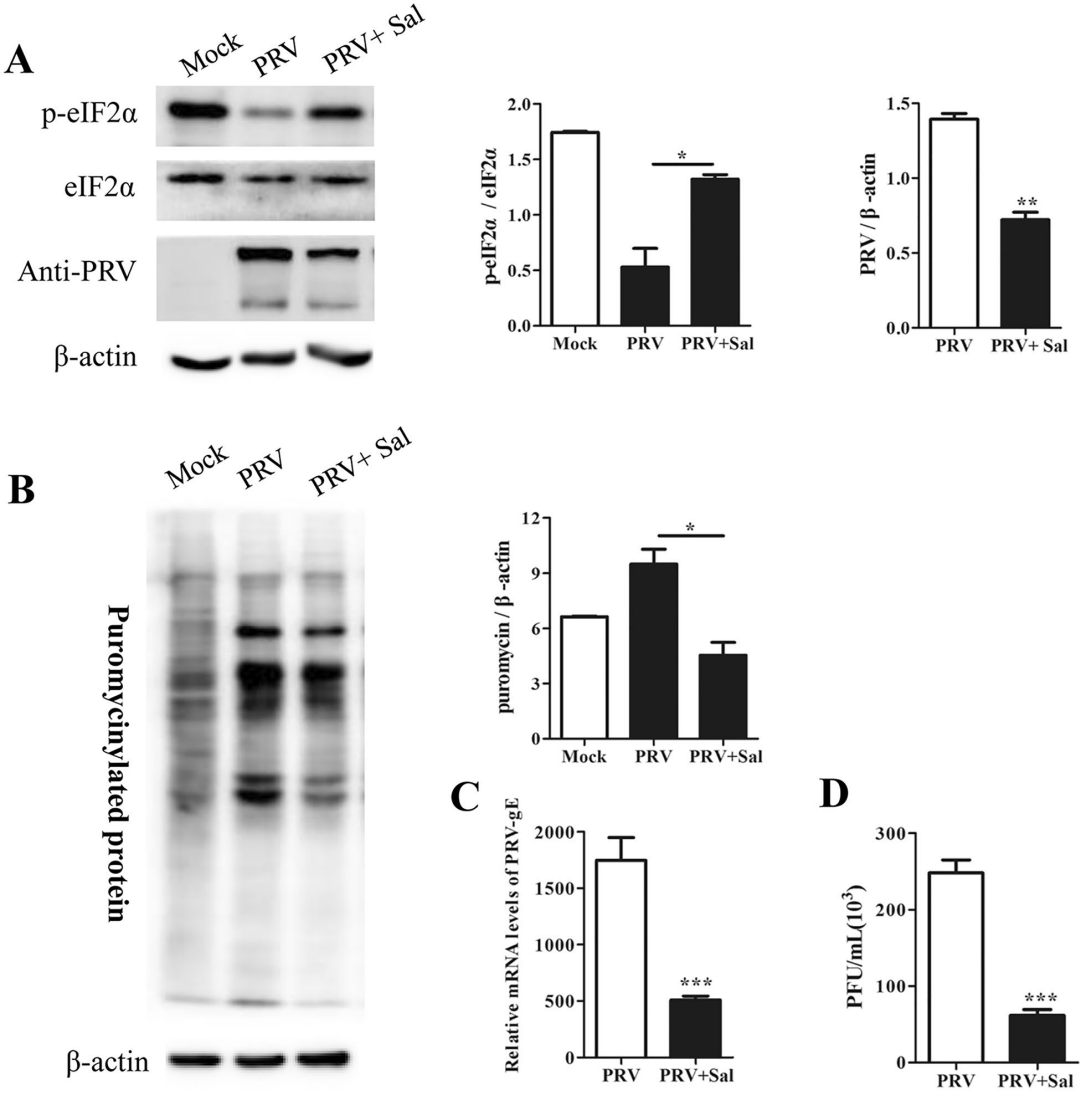
**Salubrinal increased the level of p-eIF2α and suppressed PRV replication in PK15 cells. A** PK15 cells were infected with PRV at an MOI of approximately 0.1 in the presence of 100 μM salubrinal. The cells were subjected to puromycin labelling for 1 h at 23 hpi and were then harvested at 24 hpi. Cell lysates were subjected to Western blot analysis to determine the levels of p-eIF2α, eIF2α, and PRV proteins. The intensities of the p-eIF2α bands were determined by densitometry, normalized to eIF2α, and shown as fold changes (right panel). PRV protein expression was quantified by densitometry and normalized to β-actin and are shown as fold changes (right panel). The values are presented as the mean ± SD of triplicate experiments. * *p* < 0.05; ** *p* < 0.01. **B** De novo protein synthesis was measured by using a monoclonal antibody against puromycin; the intensities of bands corresponding to puromycin-labelled proteins were quantified by densitometry and normalized to β-actin and are shown as fold changes (right panel). The values are presented as the mean ± SD of triplicate experiments. * *p* < 0.05. **C**, **D** PK15 cells were infected with PRV at an MOI of approximately 0.1 in the presence of 100 μM salubrinal for 24 h, and supernatants and cells were then harvested. RT–qPCR was performed to determine the relative mRNA level of PRV-gE compared to β-actin (**C**); the viral titre in the supernatant was determined in PK15 cells (**D**). The data are presented as the mean ± SD of three independent experiments. *** *p* < 0.001.

Many viruses modulate eIF2α phosphorylation during replication to assure viral protein synthesis and prevent cellular stress responses [7, 31]. To clarify the effect of eIF2α phosphorylation during PRV infection, PK15 cells were infected with PRV for 24 h in the presence of salubrinal (a small molecule drug that selectively blocks p-eIF2α dephosphorylation by inhibiting the PP1/GADD34 complex) [6]. Supernatants were collected, and the viral titre was determined by a plaque formation assay; in addition, the cells were labelled with puromycin and harvested for Western blot analysis. As shown in Figures 3A, B, the level of p-eIF2α was significantly increased with salubrinal treatment, whereas PRV protein levels and the global translation rate showed significant declines. Salubrinal treatment also led to significant decreases in the relative mRNA level of the PRV gE gene (encoding a glycoprotein that is important for virulence, viral spread, and intracellular signalling) and the viral titre (Figures 3C, D). These findings indicated that salubrinal treatment counteracted the PRV infection-mediated reduction in the p-eIF2α level and new protein synthesis, thereby inhibiting PRV replication.

**Figure 4:**
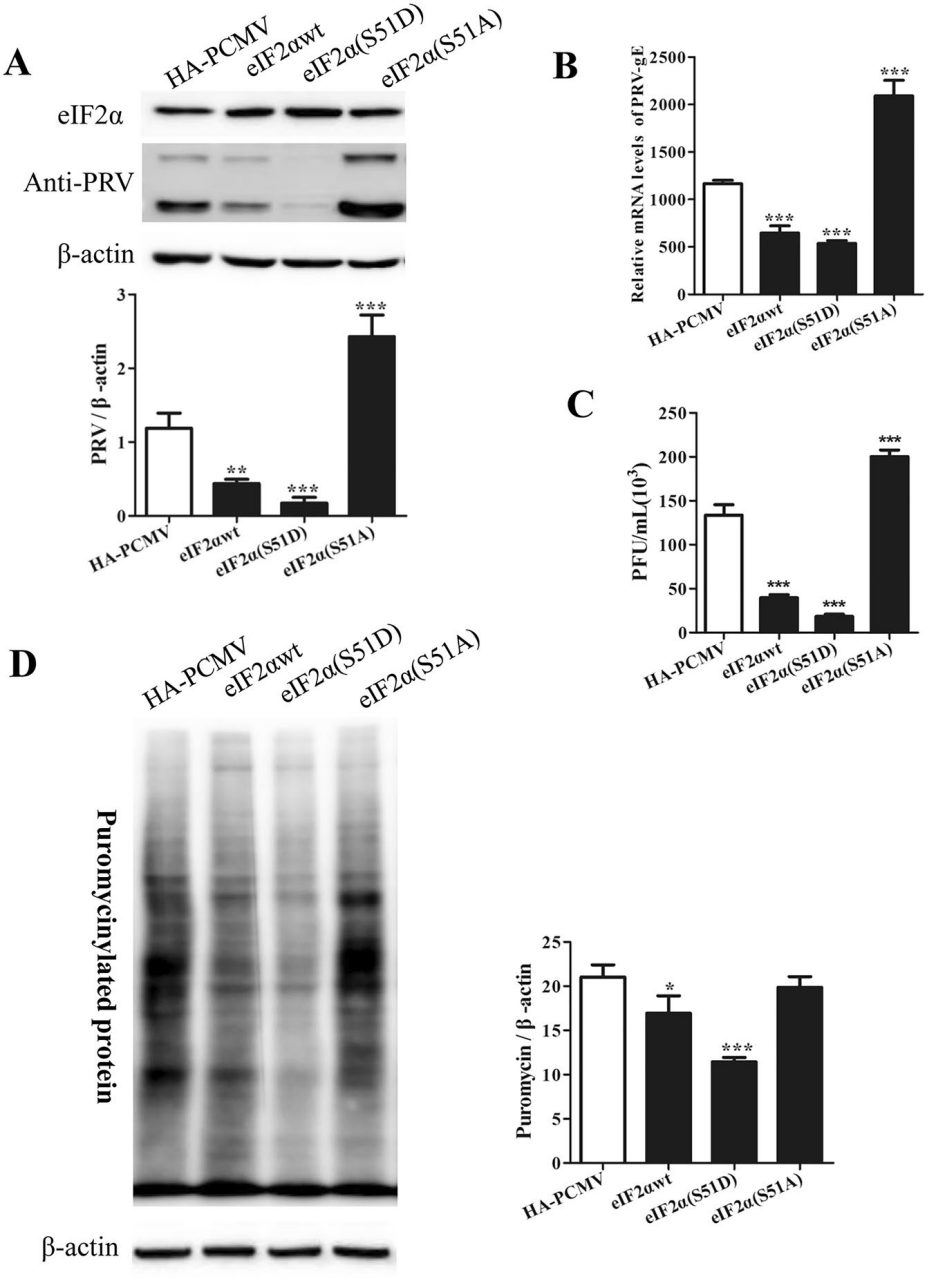
**Overexpression of eIF2αwt or eIF2α(S51D) in PK15 cells inhibited PRV replication. A** PK15 cells were transfected separately with the pCMV-HA, eIF2αwt, eIF2α(S51D), and eIF2α(S51A) constructs for 24 h and were then infected with PRV. Cells were subjected to puromycin labelling for 1 h at 23 hpi and were then harvested at 24 hpi. Western blot analysis was performed to detect eIF2α and PRV proteins. PRV protein expression was quantified by densitometry and normalized to β-actin and are shown as fold changes (bottom panel). The values are presented as the mean ± SD of triplicate experiments. ** *p* < 0.01, *** *p* < 0.001. **B**, **C** PK15 cells were transfected separately with the pCMV-HA, eIF2αwt, eIF2α(S51D), or eIF2α(S51A) constructs for 24 h and were then infected with PRV for another 24 h. Supernatants and cells were harvested. RT–qPCR was performed to determine the relative mRNA level of PRV-gE compared to β-actin (**B**); the viral titre in the supernatants was determined in PK15 cells (**C**). The data are presented as the mean ± SD of three independent experiments. *** *p* < 0.001. **D** De novo protein synthesis was measured in pCMV-HA-, eIF2αwt-, eIF2α(S51D)-, and eIF2α(S51A)-transfected cells by using a monoclonal antibody against puromycin; the intensities of bands corresponding to puromycin-labelled proteins were quantified by densitometry and normalized to β-actin and are shown as fold changes (right panel). The values are presented as the mean ± SD of triplicate experiments. * *p* < 0.05. *** *p* < 0.001.

To further elucidate the role of eIF2α phosphorylation in the replication of PRV, PK15 cells were first transfected for 24 h with constructs expressing wild-type eIF2α (eIF2αwt), a phosphomimetic form of eIF2α containing Asp instead of Ser at residue 51 [eIF2α(S51D)], or a nonphosphorylatable form of eIF2α [eIF2α(S51A)] and were then infected with PRV. The HA-PCMV vector was transfected as a control in a parallel experiment. Cells were harvested at 24 hpi for Western blot analysis, while supernatants were collected for viral titre determination by a plaque formation assay. The results showed that cells expressing either eIF2αwt or eIF2α(S51D) exhibited significantly reduced PRV replication, whereas PRV replication was restored—and even increased—in cells expressing eIF2α(S51A) (Figures 4A–C). Comparable expression levels of eIF2αwt, eIF2α(S51D), and eIF2α(S51A) were detected, as shown in Figure 4A. We subsequently determined the level of new protein synthesis in eIF2αwt-, eIF2α(S51D)-, and eIF2α(S51A)-transfected cells. The global translation rate (represented by the amount of puromycin-labelled proteins) was decreased 1.2-fold in eIF2αwt-expressing cells and 1.8-fold in cells expressing eIF2α(S51D). In contrast, the level of global protein synthesis was unaffected in eIF2α(S51A)-expressing cells (Figure 4D). These results indicated that an increase in the phosphorylation of eIF2α can repress global protein synthesis and, subsequently, PRV replication. Collectively, these data suggested that eIF2α is a target of PRV and that a high level of eIF2α phosphorylation inhibits both global and PRV infection-related protein synthesis.

### PERK and PKR phosphorylation were not affected in PRV-infected cells

**Figure 5:**
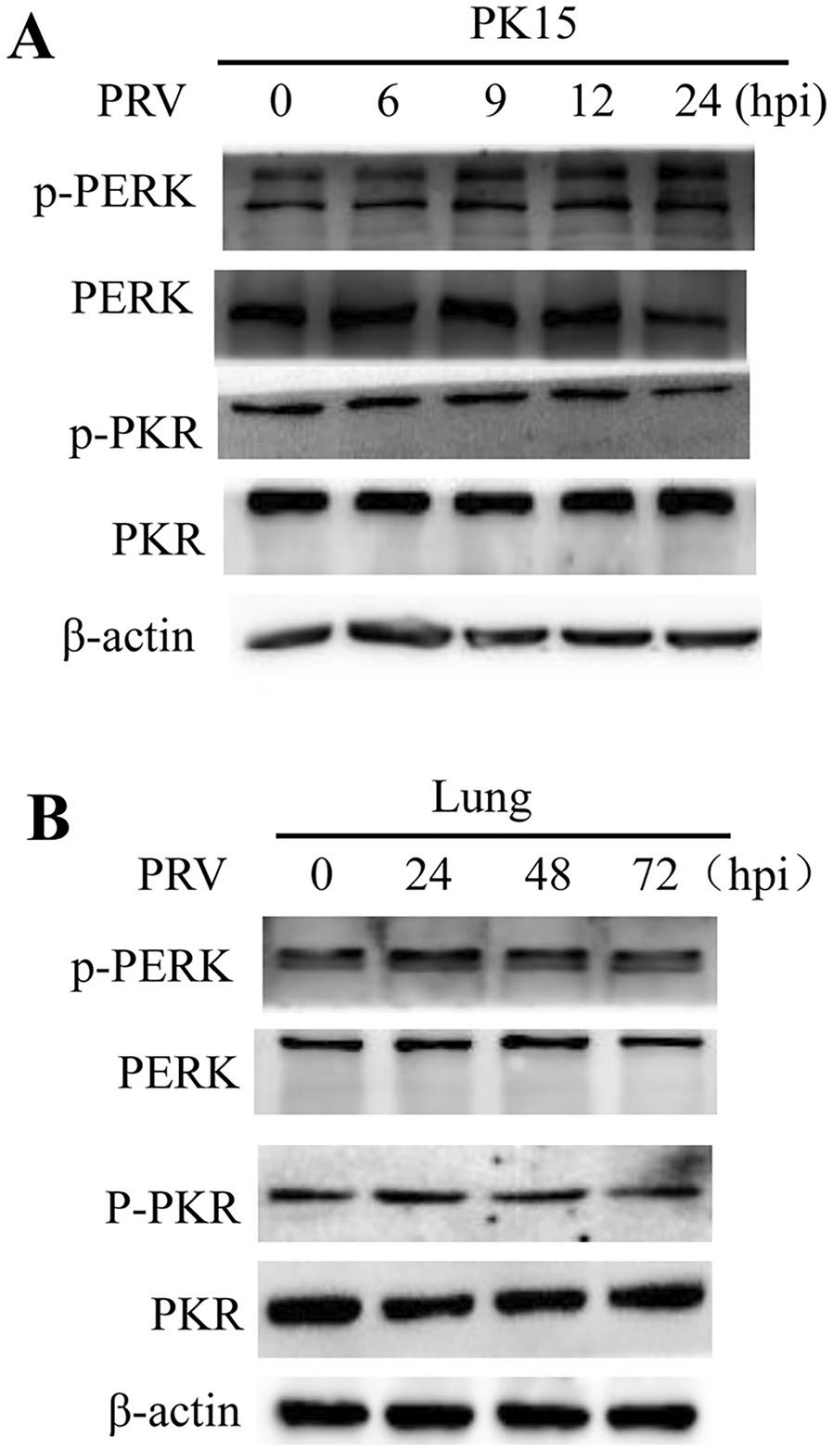
**Phosphorylation of PKR and PERK was not affected. A** PK15 cells infected with PRV at an MOI of approximately 0.1 were harvested and lysed at 0, 3, 6, 9, 12, and 24 hpi. The protein levels of p-PERK, PERK, p-PKR, and PKR were determined by Western blot analysis. **B** BALB/c mice were intraperitoneally inoculated with PRV (1 × 10^6^ PFU) and were then euthanized at 0, 24, 48, and 72 hpi. The protein levels of p-PERK, PERK, p-PKR, and PKR in the lungs were determined by Western blot analysis.

PKR and PERK belong to a family of kinases responsible for the phosphorylation of eIF2α. When activated through autophosphorylation, they phosphorylate eIF2α and, thus, are major targets of many viruses in counteracting host defence mechanisms [1, 13, 32]. To determine whether the decrease in the level of p-eIF2α was due to inhibition of PERK and PKR activation, the phosphorylation status of these two proteins was assessed in vitro and in vivo. First, virus-infected PK15 cells and the lungs of infected mice were harvested at the indicated times, and the total protein level was determined by Western blot analysis. The levels of p-PERK and p-PKR remained constant at the time points assessed both in vitro and in vivo (Figures 5A, B), suggesting that PRV infection does not influence the activation of PERK and PKR. Combined with the above results, these data indicated that the observed downregulation of eIF2α phosphorylation was not due to inhibition of PERK and PKR activation.

### PRV replication was inhibited by the protein phosphatase 1 inhibitor okadaic acid

**Figure 6:**
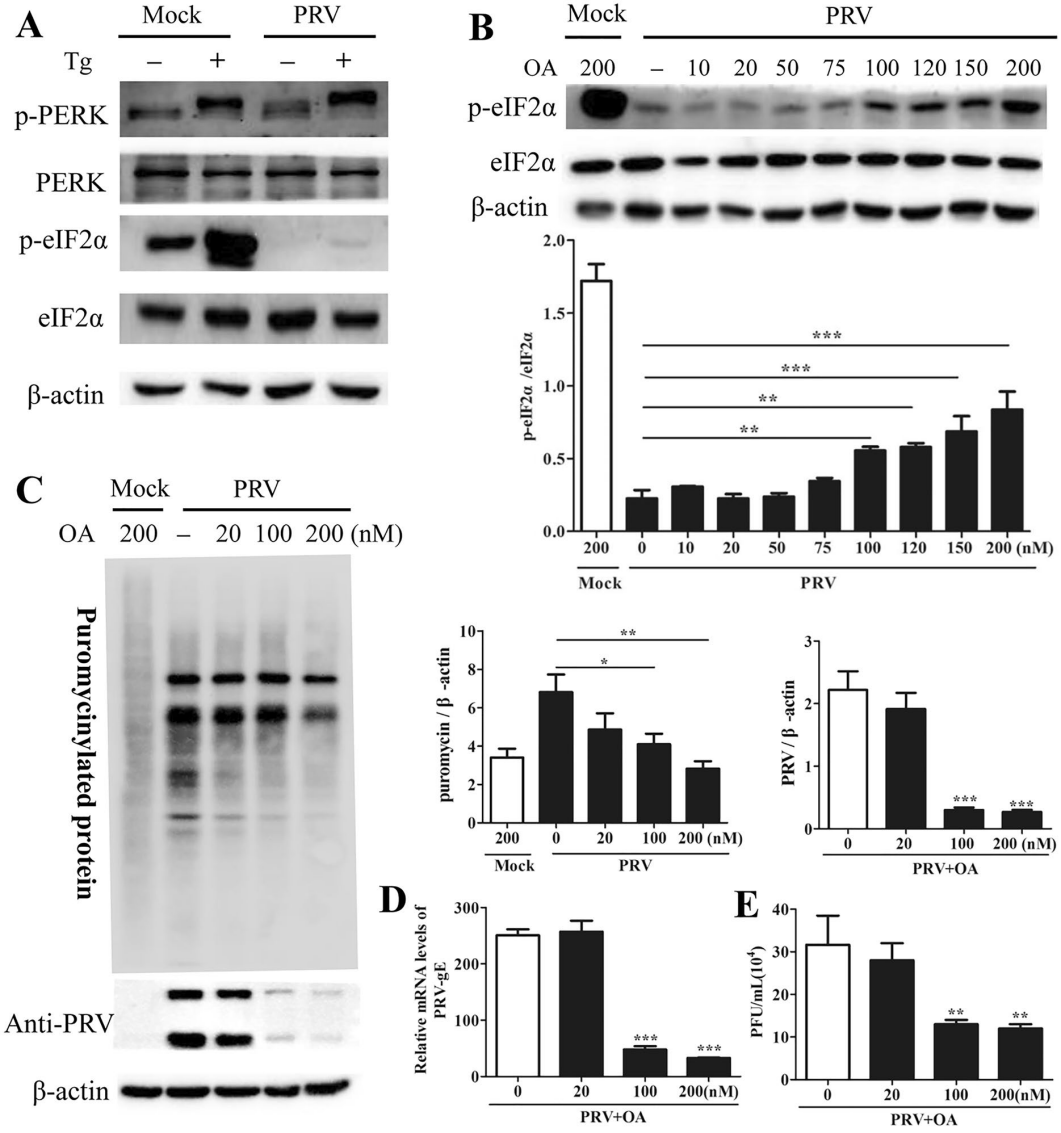
**Okadaic acid (OA) reduced PRV replication in PRV-infected PK15 cells. A** PK15 cells were infected with PRV (MOI = 0.1) in cell culture medium containing 1 µM Tg and were then harvested at 24 hpi. Western blot analysis was performed to measure p-PERK, PERK, p-eIF2α, and eIF2α protein levels. **B** PK15 cells were infected with PRV (MOI = 0.1) and incubated at 37 °C for 8 h before the addition of OA at the indicated concentrations. Cells were then incubated for another 15 h in the presence of OA, followed by puromycin labelling for 1 h. Cells were harvested at 24 hpi. Western blot analysis was performed to measure p-eIF2α, eIF2α, and PRV protein levels. The intensities of the p-eIF2α bands were determined by densitometry, normalized to eIF2α, and shown as fold changes (bottom panel). The values are presented as the mean ± SD of triplicate experiments. ** *p* < 0.01; *** *p* < 0.001. **C** De novo protein synthesis and PRV protein expression were measured by Western blot analysis; the intensities of bands corresponding to puromycin-labelled proteins were quantified by densitometry and normalized to β-actin and are shown as fold changes. The intensities of the PRV protein bands were quantified by densitometry and normalized to β-actin and are shown as fold changes (right panels). The values are presented as the mean ± SD of triplicate experiments. * *p* < 0.05; ** *p* < 0.01; *** *p* < 0.001. **D**, **E** PK15 cells were infected with PRV (MOI = 0.1) and incubated at 37 °C for 8 h before the addition of OA at the indicated concentrations. Cells were then incubated for another 16 h. Supernatants and cells were harvested at 24 hpi. RT–qPCR was performed to determine the relative mRNA level of PRV-gE compared to β-actin (**D**); the viral titre in the supernatants was determined in PK15 cells (**E**). The data are presented as the mean ± SD of three independent experiments. ** *p* < 0.01; *** *p* < 0.001.

Tg can induce eIF2α phosphorylation via the PERK-dependent UPR [33]. Here, PK15 cells were infected with PRV in the presence of Tg (1 μM) for 24 h, and Western blot analysis was performed to assess the eIF2α phosphorylation level. Tg treatment effectively induced eIF2α phosphorylation in mock-infected cells but not PRV-infected cells (Figure 6A). This finding was consistent with the results of Van Opdenbosch et al. showing that PRV is able to counteract both the preinduced (by thapsigargin) and basal levels of eIF2α phosphorylation, indicating that PRV promotes dephosphorylation of eIF2α rather than preventing its phosphorylation [29].

Protein phosphatase 1 (PP1) and protein phosphatase 2 (PP2A) have been reported to dephosphorylate eIF2α [19]. OA has been reported to inhibit the activity of type 2A phosphatases, including PP2A, PP4, and PP6, at low concentrations (IC50: 0.1–1 nM), while it inhibits the activity of type 1 phosphatases (PP1) at high concentrations (IC50: 20 nM) [28, 34–36]. PK15 cells were infected with PRV for 8 h, after which OA was added to the virus-infected cells at increasing concentrations (0 to 200 nM) for another 16 h. The results showed that low OA concentrations (10 to 75 nM) did not influence the level of p-eIF2α in PRV-infected cells, indicating that PP2A does not participate in eIF2α dephosphorylation. However, at substantially higher concentrations (100 to 200 nM), OA treatment led to a significant increase in the level of p-eIF2α (Figure 6B).

To explore the effect of OA on de novo protein synthesis and PRV replication, PRV-infected cells were treated with 0 nM OA, 20 nM OA, 100 nM OA, or 200 nM OA. Supernatants were collected for viral titre determination by a plaque formation assay, while cells (puromycin-labelled) were harvested for Western blot analysis. Both de novo protein synthesis and the synthesis of PRV proteins were decreased in cells treated with 100 nM or 200 nM OA (Figure 6C); similarly, the relative mRNA level of gE and the viral titre were significantly decreased at these concentrations of OA (Figures 6D, E). Furthermore, 20 nM OA treatment did not influence global protein synthesis or PRV replication. These results suggested that suppression of PP1 activity inhibits de novo protein synthesis and PRV replication.

Collectively, these findings demonstrated that suppressing PP1 activity could inhibit PRV replication via attenuation of the host’s translational machinery by increasing the eIF2α phosphorylation level.

### GADD34 was involved in eIF2α dephosphorylation and PRV replication

**Figure 7:**
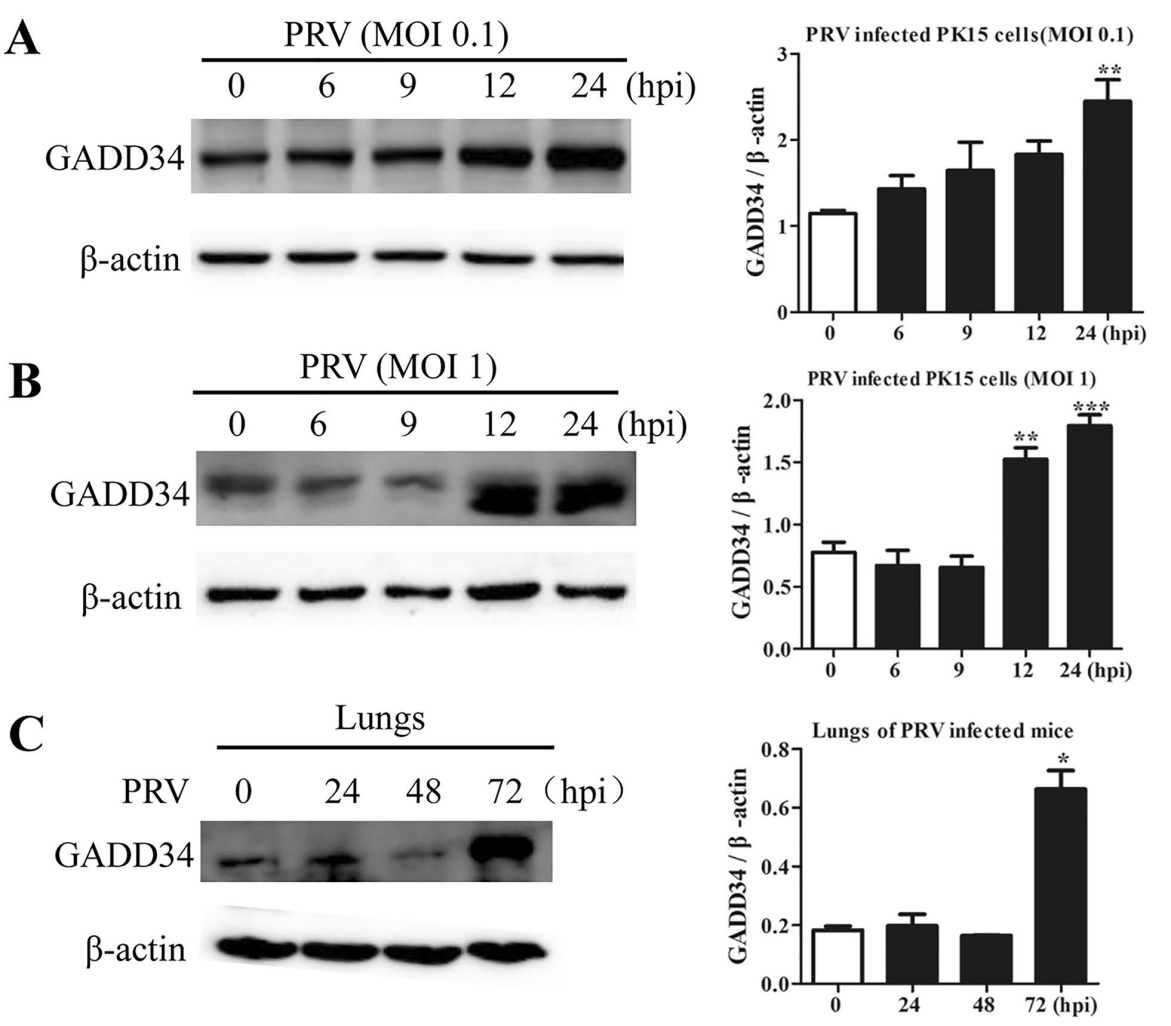
**PRV infection induced the expression of GADD34 in vitro and in vivo.** PK15 cells were infected with PRV at an **A** MOI of 0.1 or **B** MOI of 1.0 at 0, 6, 9, 12, and 24 hpi. Then, the protein level of GADD34 was determined by Western blot analysis; the intensities of the GADD34 bands were quantified by densitometry and normalized to β-actin and are shown as fold changes (right panel). The values are presented as the mean ± SD of triplicate experiments. ** *p* < 0.01; *** *p* < 0.001. **C** BALB/c mice were intraperitoneally inoculated with PRV (1 × 10^6^ PFU) and were then euthanized at 0, 24, 48, and 72 hpi. The protein level of GADD34 in the lungs was determined by Western blot analysis; the intensities of the GADD34 bands were quantified by densitometry and normalized to β-actin and are shown as fold changes (right panel). The values are presented as the mean ± SD of triplicate experiments. * *p* < 0.05.

**Figure 8:**
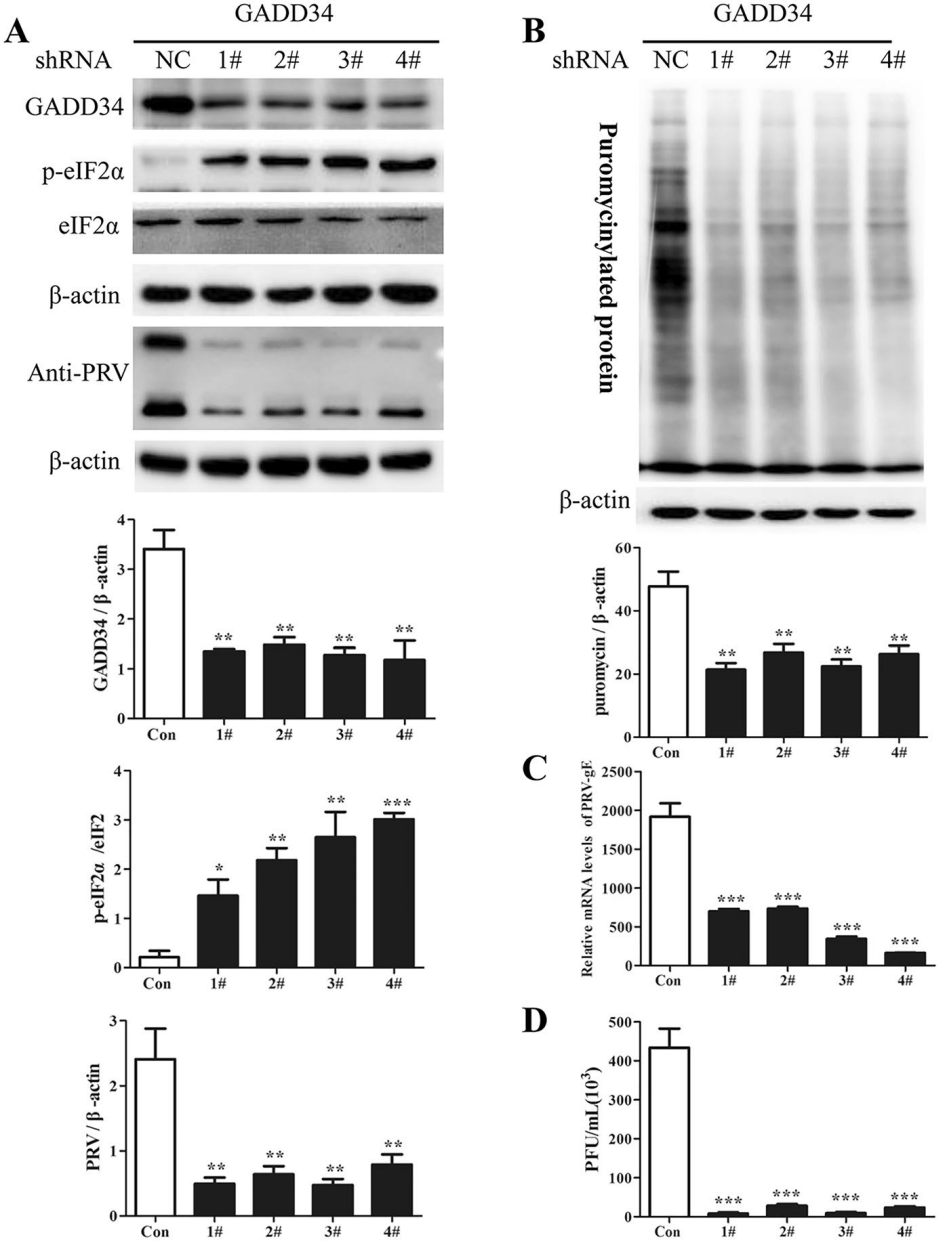
**Knockdown of GADD34 in PK15 cells inhibited PRV replication. A** PK15 cells were transfected with shNC or shGADD34 (#1, #2, #3, and #4) for 24 h and were then infected with PRV (MOI = 0.1). Cells were subjected to puromycin labelling for 1 h at 23 hpi and were then harvested at 24 hpi. Western blot analysis was performed to detect p-eIF2α, eIF2α, and PRV proteins; GADD34 and PRV protein expression was quantified by densitometry and normalized to β-actin and are shown as fold changes. The intensities of the p-eIF2α bands were quantified by densitometry, normalized to eIF2α, and shown as fold changes (bottom panels). The values are presented as the mean ± SD of triplicate experiments. * *p* < 0.05; ** *p* < 0.01; *** *p* < 0.001. **B** De novo protein synthesis was measured in GADD34 knockdown cells by using a monoclonal antibody against puromycin; the intensities of bands corresponding to puromycin-labelled proteins were quantified by densitometry and normalized to β-actin and are shown as fold changes (the lower panel). The values are presented as the mean ± SD of triplicate experiments. ** *p* < 0.01. **C**, **D** PK15 cells were transfected with shNC or shGADD34 (#1, #2, #3, and #4) for 24 h and were then infected with PRV (MOI = 0.1). Supernatants and cells were harvested at 24 hpi. RT–qPCR was performed to determine the relative mRNA level of PRV-gE compared to β-actin (**C**); the viral titre in the supernatants was determined in PK15 cells (**D**). The data are presented as the mean ± SD of three independent experiments. *** *p* < 0.001.

GADD34 promotes translational recovery by recruiting PP1 to dephosphorylate eIF2α, thus restoring global protein synthesis; however, many viruses are capable of regulating this process [21, 37]. To determine whether PRV infection influences GADD34 protein expression, PK15 cells were infected with PRV at different MOIs (0.1 or 1) and were then harvested at the indicated times for Western blot analysis. As shown in Figure 7A, the protein level of GADD34 increased at 24 hpi at an MOI of 0.1, while it increased significantly from 12 to 24 hpi at an MOI of 1 (Figure 7B). Similarly, PRV infection induced obvious expression of GADD34 in the lungs of mice at 72 hpi (Figure 7C). To further investigate the role of GADD34 in eIF2α phosphorylation and PRV replication, the levels of eIF2α phosphorylation and PRV replication were examined in cells with shRNA-mediated GADD34 knockdown. The knockdown efficiency of GADD34 shRNA was confirmed by immunoblotting for GADD34 (Figure 8A). The results showed that the level of p-eIF2α was increased in GADD34-depleted cells, whereas the levels of PRV proteins were decreased (Figure 8A). Moreover, both the relative mRNA level of gE and the viral titre decreased significantly with GADD34 knockdown (Figure 8C, D), as did global protein synthesis (Figure 8B). Collectively, these results indicated that PRV may induce upregulation of GADD34, thereby promoting eIF2α dephosphorylation and restoring global translation, with a consequent increase in viral protein synthesis.

## Discussion

Viruses lack biosynthetic capabilities and must coopt the host cell translational machinery to produce progeny virions [37]. Consequently, many viruses have evolved a variety of regulatory mechanisms to coerce the cellular translation machinery into synthesizing viral proteins. Among these mechanisms, one of the most extensively studied is phosphorylation/dephosphorylation of eIF2α, with modulation of this pathway (reduced phosphorylation/increased dephosphorylation) resulting in preferential expression of viral gene products [38]. IBV and Zika virus (ZIKV) markedly suppress eIF2α phosphorylation, which benefits viral replication [28]. HSV-1, also a herpesvirus, suppresses eIF2α phosphorylation, thereby preventing global translation attenuation and ensuring that abundant viral mRNAs are translated in cells after viral DNA replication [39]. In this study, we found that global protein synthesis was increased during PRV replication. In addition, eIF2α phosphorylation was significantly decreased and was even barely detectable. Similarly, Van Opdenbosch et al. reported that the PRV Becker strain efficiently suppresses eIF2α phosphorylation during the early stages of viral replication [29]. In the present study, we detected the progression of protein synthesis and eIF2α phosphorylation in cells infected with PRV at different MOIs (0.1 and 1). When cells were infected with PRV at an MOI of 0.1, an increased level of protein synthesis and a reduced level of eIF2α phosphorylation were detected until 12 hpi, while in cells infected at an MOI of 1, an increase in protein synthesis and a reduction in eIF2α phosphorylation were detected at 9 hpi. Infection at MOIs of 0.1 and 1 elicited different results in terms of the temporal changes in the levels of de novo protein synthesis and eIF2α phosphorylation, suggesting that protein synthesis and eIF2α phosphorylation are altered along with viral replication. Importantly, the p-eIF2α level in lung tissues of mice also exhibited a significant decrease during PRV infection.

Phosphorylation of eIF2α attenuates global translation by inhibiting the delivery of the initiator Met-tRNAi to the initiation complex [40]. Therefore, it is not surprising that increased phosphorylation of eIF2α may adversely affect viral infection. Indeed, treating ZIKV-infected cells with salubrinal leads to accumulation of p-eIF2α and inhibition of viral replication [31], while salubrinal treatment inhibits HSV replication in a dose-dependent manner and results in a higher level of p-eIF2α [41]. Furthermore, an increase in the level of phosphorylated eIF2α via eIF2α overexpression inhibits translation and suppresses IBV infection [28]. Similarly, in the present study, global translation and PRV replication were greatly impaired in salubrinal-treated cells, eIF2αwt-expressing cells, and eIF2α(S51D)-expressing cells. These observations indicate that eIF2α is a target of PRV, while eIF2α phosphorylation inhibits protein synthesis and, consequently, PRV infection.

Phosphorylation of eIF2α is modulated by the opposing activities of kinases and phosphatases [7]. PKR and PERK are two key eIF2α kinases and are, therefore, major targets of many viruses in attempts to counteract host defence mechanisms [1]. IBV infection greatly reduces the level of phosphorylated PKR, which results in suppression of eIF2α phosphorylation and facilitates IBV replication [28]. In this study, the levels of p-PERK and p-PKR did not change in response to PRV infection either in vitro or in vivo, indicating that the PRV-induced reduction in eIF2α phosphorylation is independent of the suppression of PERK and PKR activation. However, PRV efficiently countered eIF2α phosphorylation in Tg-stimulated cells, in addition to reducing the basal level of eIF2α phosphorylation. Similar results have been reported in PRV Becker strain-infected rat 50B11 neuronal cells and swine testicle cells [29], suggesting that PRV infection leads to dephosphorylation of eIF2α rather than preventing its phosphorylation. OA is a potent inhibitor of type 1 and 2A phosphatases [42]. In the present study, we found that OA treatment inhibited the host’s translational machinery, thereby inhibiting PRV replication by suppressing PP1 activity and, consequently, increasing eIF2α phosphorylation. In line with our current results, Van Opdenbosch et al. also observed involvement of PP1 in PRV-mediated inhibition of eIF2α phosphorylation. Indeed, as in the current manuscript, they found that a low concentration of OA (20 nM) did not affect PRV-mediated eIF2α dephosphorylation, whereas a PP1 inhibitor suppressed this process [29].

GADD34 is induced by stresses such as viral infection and forms a complex with PP1 that specifically promotes eIF2α dephosphorylation [21]. IBV can induce the expression of GADD34, whereas knockdown of GADD34 increases the eIF2α phosphorylation level and delays IBV replication [28]. In the present study, we found that PRV induced the expression of GADD34 both in vitro and in vivo. Additionally, in GADD34-depleted cells, the level of p-eIF2α was increased, whereas global translation and PRV replication were reduced. This suggests that PRV induces GADD34 expression to promote eIF2α dephosphorylation, thereby maintaining viral protein synthesis. It has been reported that ATF-4 (also called CREB-2) enters the nucleus to activate the transcription of GADD34, thereby enhancing eIF2α dephosphorylation and restoring global translation [43]. Consequently, further studies are needed to explore whether ATF4 participates in regulating GADD34 expression during PRV infection. Furthermore, both knockdown of GADD34 and OA treatment only partially restored the level of p-eIF2α during PRV infection, implying that multiple cellular pathways and viral components may influence the eIF2α phosphorylation status. Some viruses encode a protein that mimics the function of GADD34. For instance, HSV encodes a protein (ICP34.5) that is homologous to GADD34 and binds to PP1, thereby promoting eIF2α dephosphorylation and ensuring viral replication [44, 45]. The DP71L protein of African swine fever virus shares sequence similarity with the C-terminal domain of the HSV ICP34.5 protein and GADD34 and can promote eIF2α dephosphorylation and restore protein synthesis during viral infection [46]. In addition, nonstructural protein 7 from transmissible gastroenteritis virus interacts with PP1, thereby mediating eIF2α dephosphorylation [47].

In conclusion, our data demonstrated that PRV manipulates GADD34 expression to negatively regulate eIF2α phosphorylation, which is beneficial for de novo protein synthesis and PRV propagation.

## Data Availability

The datasets used and/or analysed during the current study are available from the corresponding author on reasonable request.
